# Renal Safety of Distal Renal Denervation on Kidney Function in Diabetic Patients with Resistant Hypertension

**DOI:** 10.3390/medicina62020274

**Published:** 2026-01-28

**Authors:** Musheg Manukyan, Victor Mordovin, Stanislav Pekarskiy, Irina Zyubanova, Valeria Lichikaki, Ekaterina Solonskaya, Simzhit Khunkhinova, Anna Gusakova, Alla Falkovskaya

**Affiliations:** Cardiology Research Institute, Tomsk National Research Medical Center, Russian Academy of Sciences, 634050 Tomsk, Russia; mordovin@cardio-tomsk.ru (V.M.); pekarski@cardio-tomsk.ru (S.P.); ziv@cardio-tomsk.ru (I.Z.); lichikaki@cardio-tomsk.ru (V.L.); tsoy@cardio-tomsk.ru (E.S.); hsa@cardio-tomsk.ru (S.K.); anna@cardio-tomsk.ru (A.G.); alla@cardio-tomsk.ru (A.F.)

**Keywords:** renal denervation, renal function, renal hemodynamics, type 2 diabetes mellitus, resistant hypertension, radiofrequency ablation, blood pressure monitoring

## Abstract

*Background and Objectives:* The combination of resistant hypertension (RHTN) and type 2 diabetes mellitus (T2DM) accelerates the development of chronic kidney disease (CKD), which may be largely associated with sympathetic hyperactivity. Distal renal denervation (dRDN) effectively reduces sympathetic flow to the kidneys, causing renal vasodilation and increased renal perfusion. However, this effect may be limited by nephrotoxicity due to the multiple increase in the number of contrast injections, as well as a significant blood pressure (BP) reduction, which naturally worsens renal perfusion. This study aimed to test the hypothesis that dRDN prevents the progressive decline in kidney function in patients with RHTN and T2DM. *Materials and Methods:* The prospective interventional study (REFRAIN, NCT04948918) included men and women > 20 y.o. with true RHTN. Eligible patients underwent dRDN. The primary endpoint was a change in eGFR from baseline to 12 months. Secondary endpoints were changes in 24 h BP, serum lipocalin-2, cystatin C, 24 h urinary albumin excretion, renal blood flow, and kidney volumes (by MRI). Multiple regression analysis was used to find independent predictors of individual estimated glomerular filtration rate (eGFR) change. *Results:* A total of 29 patients with RHTN and T2DM were included in the study (61.6 ± 7.2 y.o., 10 males, mean 24 h ambulatory BP: 158.1 ± 21.4/81.8 ± 12.4 mmHg (systolic/diastolic, respectively)), HbA1c: 7.8 ± 1.4%, and eGFR 56.7 ± 19.9 mL/min/1.73 m^2^, 23 (79%) patients with CKD, and 2 patients with albuminuria only. There were no perioperative complications. Twenty-seven (93%) participants completed 12 month follow-up. eGFR did not change from baseline: +1.3 mL/min/1.73 m^2^ [95% CI: −9.6, 12.1], despite the expected decrease due to a significant decrease in 24 h systolic BP (−18.2 mmHg [95% CI: −28.6, −7.8]). No changes in other secondary endpoints were observed. Independent predictors of individual eGFR change were baseline 24 h pulse pressure (*p* = 0.030) and HbA1c (*p* = 0.010). *Conclusions:* Distal RDN demonstrates a substantial nephroprotective effect in patients with RHTN and T2DM, which may be partly mediated by a reduction in arterial stiffness and is negatively dependent on baseline hyperglycemia.

## 1. Introduction

The prevalence of hypertension (HTN) and type 2 diabetes mellitus (T2DM) continues to steadily increase [1], significantly contributing to the growing burden of chronic kidney disease (CKD) [2,3,4]. By 2040, CKD is projected to become the fifth leading cause of disability-adjusted life years worldwide [5]. On 23 May 2025, the World Health Assembly (WHA78) adopted its first ever resolution focused exclusively on kidney health [6]. Patients with resistant hypertension (RHTN) have particularly poor renal outcomes [7]. At the same time, the development of CKD itself makes it difficult to control blood pressure (BP), which closes the vicious circle between HTN and CKD, among which 40% of patients have RHTN [8]. Moreover, as renal function declines, the risk of developing RHTN increases by 1.6-fold in CKD stage G3a and doubles in more advanced CKD stages [9]. Importantly, T2DM not only increases CKD risk, but also contributes to antihypertensive treatment resistance [10], partially through renal injury mechanisms. Both HTN and T2DM lead to progressive renal function decline and represent major causes of CKD. According to Polonia et al., patients with HTN or T2DM experience an annual estimated glomerular filtration rate (eGFR) decline of 2–4 mL/min/1.73 m^2^ [11]. Furthermore, therapeutic BP reduction naturally decreases eGFR due to the dependence of the latter on filtration pressure, a factor that must be considered when evaluating treatment effects. Thus, the assessment and interpretation of the effects of an antihypertensive treatment on kidney function in patients with HTN and T2DM should take both of these phenomena into account. That is, the null hypothesis for detecting the effect of antihypertensive treatment on kidney function in patients with HTN and T2DM is not a zero change but a non-zero decrease predicted from the known rate of the function decline over time and actual BP lowering effect of the treatment. Likewise, to calculate the magnitude of such an effect, the reference level should be taken, for example, not just as the pre-treatment level, but the pre-treatment level minus the reduction expected as a result of the natural progression of renal dysfunction over the given time period and minus the reduction expected as a result of the decrease in BP from the baseline.

The pathogenesis of HTN and T2DM shares common mechanisms related to the hyperactivity of the sympathetic nervous system (SNS) playing a pivotal role in the disease progression and development of adverse outcomes [12,13], especially renal dysfunction. Excessive stimulation of the adrenoceptors in the kidneys causes renal vasoconstriction and thus the reduction in blood oxygen supply to the renal tissue while also enhancing the sodium reabsorption associated with high oxygen consumption. In combination, these effects can cause severe renal tissue hypoxia, extensively shown as a major cause of CKD. A direct correlation exists between an increased sympathetic tone and reduced eGFR [12]. Consequently, SNS has emerged as a therapeutic target not only for HTN but also for CKD, driving the development of neuromodulation technologies, e.g., renal denervation. Renal denervation (RDN) reduced sympathetic hyperactivity [14] and BP [15] and affects the key pathophysiological mechanisms of RHTN and CKD [16,17]. Specifically, RDN blocks the transmission of excessive sympathetic stimuli to the kidneys, which causes sustained vasodilation with a significant increase in renal perfusion, while it simultaneously reduces sodium reabsorption and the associated high oxygen consumption. The restoration of renal tissue oxygenation terminates hypoxic damage to the renal tissue and the associated progression of renal dysfunction in patients with HTN and T2DM, including those who already have CKD.

The potential nephroprotective effect of RDN has been demonstrated in several studies, including those with T2DM [18,19,20,21,22]. Moreover, patients with CKD may derive even greater benefit compared to those without renal impairment [23]. Currently, renal denervation is included as a therapeutic option for the management of HTN [16,17]. However, most available evidence comes from procedures performed using anatomically suboptimal ablation techniques targeting the main renal artery trunk. Distal renal denervation (dRDN) is the latest version of the therapy, optimized according to the actual anatomical shape of the renal nerve plexuses. It demonstrated a more pronounced antihypertensive effect and thus was expected to have a more powerful nephroprotective effect. However, the nephroprotective potential of dRDN may be limited by the increased nephrotoxicity due to the multiple increase in the number of contrast injections, since multiple segmental branches are treated instead of a single renal artery trunk, as well as by the significant BP lowering effect, which naturally compromises renal perfusion.

Therefore, the aim of this study was to test the hypothesis that dRDN prevents progressive kidney function decline in patients with RHTN and T2DM.

## 2. Materials and Methods

A single-center prospective interventional study with a 12 month follow-up period was conducted at the Research Institute of Cardiology, Tomsk National Research Medical Center (the corresponding study protocol, titled “Distal Renal Denervation to Prevent Renal Function Decline in Patients With T2DM and Hypertension”, REFRAIN, is registered at ClinicalTrials.gov under identifier NCT04948918). Patients were screened and enrolled from January 2020 to December 2022. All patients provided written informed consent prior to inclusion in the study.

Eligible patients met the following inclusion criteria: (1) men and women, 20–80 years old, (2) diagnosis of T2DM (glucose tolerance test > 11.0 mmol/L, glycated hemoglobin (HbA1c) > 6.5%), (3) office systolic blood pressure (SBP) ≥ 140 mmHg, while receiving three or more antihypertensive medications (one is a diuretic) without changes for 3 months prior to enrollment.

The exclusion criteria were as follows: (1) pseudo-resistant HTN, (2) secondary HTN, (3) HbA1c > 10%, (4) eGFR < 30 mL/min/1.73 m^2^, (5) pregnancy, (6) renal artery anatomy ineligible for treatment, (7) severe comorbidity significantly increasing the risk of the intervention according to investigator’s assessment.

Renal dysfunction was defined as an eGFR < 60 mL/min/1.73 m^2^, or repeated albuminuria ≥ 30 mg/g creatinine in the spontaneous urine (G3 or G2A2 by KDIGO 2012) [24].

Office BP measurement according to standard practice and 24 h BP monitoring was performed at baseline and 1 year after the procedure. Variations in the concomitant antihypertensive drug therapy were assessed by the change in the average number of medications taken according to the interview.

Laboratory parameters of renal function were evaluated in all patients included in the study baseline and 1 year after the procedure: serum creatinine, eGFR by using the CKD-EPI formula, serum Lipocalin-2 (neutrophil gelatinase-associated lipocalin (NGAL) (Human Lipocalin 2 ELISA test system (BioVendor Laboratory Medicine, Inc., Brno, Czech Republic)), cystatin C blood levels (BioVendor Laboratory Medicine, Inc., Brno, Czech Republic), (free metanephrine and normetanephrine in human plasma (MetCombi Plasma ELISA kit (IBL International GMBH, Hamburg, Germany)), HbA1c, 24 h urine volume, 24 h urinary excretion of albumin, potassium and sodium, metanephrine, and normetanephrine. 

Renal Doppler flowmetry with calculation of renal resistive index (RRI) [25] and kidney magnetic resonance imaging (MRI) (1.5 Tesla, Titan vantage, Toshiba, Otawara, Japan) were performed at baseline and 1 year after RDN. An average value of the RRI was used from the upper, middle, and lower segments of the kidney. A detailed description of the MRI protocol was published elsewhere [26].

### 2.1. Renal Denervation Procedure

**Figure 1:**
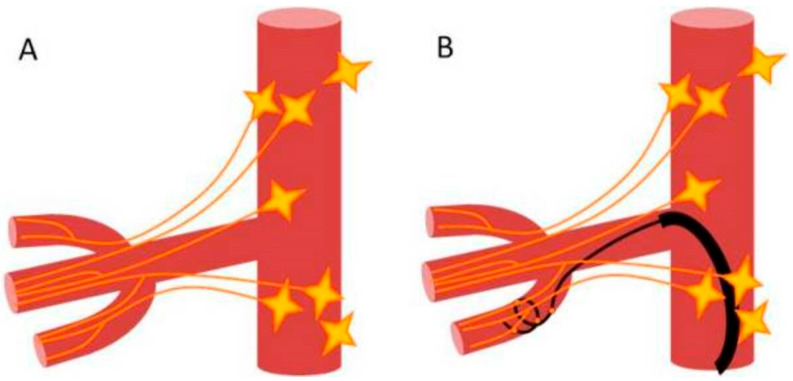
Actual anatomy of the renal nerve plexus (**A**). Anatomically optimized distal renal denervation (**B**).

The dRDN was performed using the 6 F Symplicity Spyral catheter, which was sequentially advanced into segmental branches of the renal arteries where the 4 electrodes were deployed in a pre-defined helical pattern and radiofrequency energy was delivered simultaneously to all 4 quadrants of arterial wall circumference in a spiral fashion (Figure 1). The two such treatments in different positions were performed in each segmental branch. If the branch was too short, the treatment was applied across the bifurcation.

### 2.2. Study Endpoints

The primary study outcome was the change in eGFR from baseline to 12 months.

The secondary outcomes included efficacy and safety endpoints. Efficacy endpoints were changes after 12 months in the following: office BP levels (systolic/diastolic); 24 h BP (24 h mean, daytime, nighttime; systolic/diastolic); cystatin C blood levels; NGAL blood levels; 24 h urinary albumin excretion urinalysis; cortical and medullary volume of the kidneys and their ratio according to MRI (cortical and medullary volume measured using magnetic resonance imaging); RRI in a trunk and segmental renal arteries; peak linear blood flow velocity in the trunk and in segmental renal arteries blood. The safety endpoints were acute kidney injury incidence and major adverse renal events: the new-onset kidney injury (persistent albuminuria/proteinuria and/or sustained reduction in eGFR to <60 mL/min/1.73 m^2^), progression to end-stage kidney disease (eGFR < 15 mL/min/1·73 m^2^), and renal-related mortality (death attributable to kidney failure or dialysis-associated complications) were assessed over the entire study period.

The secondary analysis encompassed a comparative evaluation of patient subgroups stratified by the magnitude of antihypertensive responses to the intervention, alongside an exploration of determinants modulating renal functional changes following dRDN. Given that renal perfusion is intrinsically dependent on systemic BP, and excessive BP reduction may compromise kidney function [27], a supplementary analysis was performed. Patients were retrospectively stratified into responders and non-responders based on the magnitude of BP reduction at 12 months after dRDN. Responders were defined as individuals achieving a ≥10 mmHg reduction in 24 h ambulatory systolic BP (24 h SBP). Furthermore, responders were subclassified into two subgroups: moderate responders (10–20 mmHg reduction in 24 h SBP) and super-responders (>20 mmHg reduction in 24 h SBP).

A multiple linear regression analysis was performed to find the factors influencing the change in renal function after dRDN. The model included HbA1c, age, sex, baseline eGFR, and 24 h SBP as undependent variables whereas eGFR change was used as the dependent variable.

The study was carried out using equipment from the Medical Genomics Center of Collective Use of the Tomsk National Research Medical Center.

### 2.3. Statistical Analysis

Statistica 10.0 ver. for Windows and SPSS 26 were used for the statistical analysis. The significance of differences in categorical variables was tested using Fisher’s exact test. The Shapiro–Wilk test was used to test the hypothesis of a normal distribution of continuous variables. Between-group differences were tested using unpaired *t*-tests for normally distributed variables and the Mann–Whitney U test otherwise. Within-group differences in repeated measures were assessed using paired *t*-test. The association between variables was assessed using Pearson’s correlation coefficient (r). Multiple linear regression analysis was performed to identify predictors of the development of the analyzed effects. The 95% confidence intervals (CIs) were calculated to assess the size of the treatment effects according to the International Conference on Harmonization E9 Guideline: “Statistical Principles for Clinical Trials”. Analyses were performed based on the per-protocol principle. Statistical significance was set at *p* < 0.05.

## 3. Results

### 3.1. Baseline Characteristics

The baseline clinical and demographic characteristics of the study cohort are presented in Table 1. The overall cohort comprised patients aged over 60 years, with a female predominance. The majority have both general and abdominal obesity, along with comorbid coronary artery disease and peripheral atherosclerosis. Nephropathy was documented in 79% of patients.

**Figure 2:**
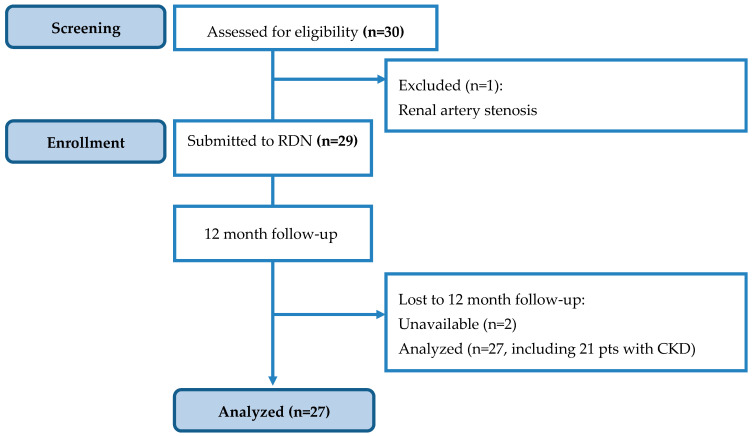
Study flow chart. From the total number of patients (n = 30), 29 patients underwent renal denervation, after the exclusion of one patient due to renal artery stenosis.

A total of 29 subjects were finally enrolled in the study and underwent dRDN. Figure 2 summarizes the recruitment process and patient journey.

Baseline antihypertensive and concomitant medication prescription is shown in Table 2. All patients were taking statins.

### 3.2. Safety

The technical success of the procedure (at least four full-time applications of radiofrequency energy performed on each side and mean impedance drop of ≥5%) was 100%. Intraprocedural angiography revealed no significant vascular injuries to the renal arteries or their branches, and no allergic reaction to the contrast agent. No safety concerns, perioperative complications (bleeding, hematoma, dissection, or perforation), or instances of acute kidney injury were observed during the study period.

### 3.3. Primary Endpoint Analysis

No significant changes in eGFR were observed in the study group at one year post-dRDN compared to baseline (Δ = 1.3 mL/min/1.73 m^2^ [95% CI: −9.6; 12.1], *p* = 0.824).

### 3.4. Secondary Endpoint Analysis

#### 3.4.1. Assessment of Antihypertensive Efficacy

**Figure 3:**
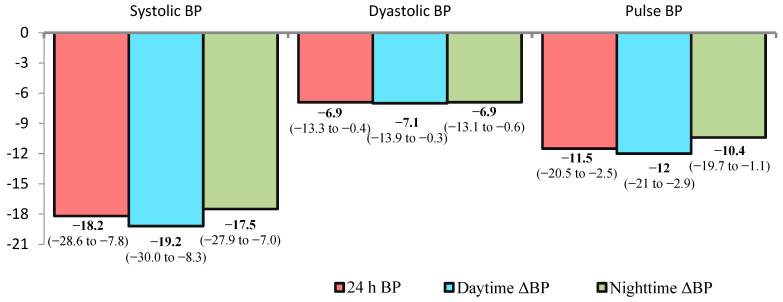
Change in ambulatory BP at 12 months after RDN compared to baseline. Data are mean and 95% confidence interval.

At 12 months after dRDN, significant reductions in office BP, 24 h ambulatory BP, pulse pressure (PP), and systolic BP variability were observed (Figure 3). The frequency of elevated pulse pressure (>60 mmHg) decreased by 1.9-fold, from 79% (n = 23) to 41% (n = 12) (*p* = 0.007).

Target office BP levels (<140/90 mmHg) were achieved in 12 patients (44%). The analysis of changes in structural–functional renal parameters is presented in Table 3.

#### 3.4.2. Retrospective Comparison of Outcomes in Groups with Mild and Strong BP Lowering Effect

The proportion of responders (defined as ≥10 mmHg reduction in 24 h systolic BP) was 63% (n = 17). Among these, 11 patients (41%) achieved a >20 mmHg reduction in 24 h systolic BP, constituting the super-responder subgroup. The frequency of elevated PP (>60 mmHg) decreased by 1.9-fold, declining from 79% (n = 23) to 41% (n = 12) (*p* = 0.007). Subgroup analyses comparing responders and non-responders are presented in Table 4. No significant differences in these parameters were observed between the subgroups.

It is noteworthy that the proportion of patients with CKD was comparably high in both responder and non-responder groups (n = 14 [82.4%] vs. n = 7 [70%], respectively). No significant differences in eGFR changes were observed based on the magnitude of BP reduction—categorized as 10–20 mmHg (moderate responders) or >20 mmHg (super-responders) (−0.6 ± 11.4 vs. −2.4 ± 12.5, *p* = 0.758). Given the study population (patients with DM) and the sympatholytic mechanism of the intervention, we further evaluated trends in glucose metabolism and catecholamine levels (Table 5). No significant alterations in these parameters were detected.

#### 3.4.3. Predictors of Kidney Function Changes Following Distal RDN 

Multivariate regression analysis (Table 6) identified baseline HbA1c and PP as independent predictors of eGFR changes at 12 months post-RDN. Lower baseline HbA1c and PP levels were associated with greater improvement in eGFR following the intervention.

**Figure 4:**
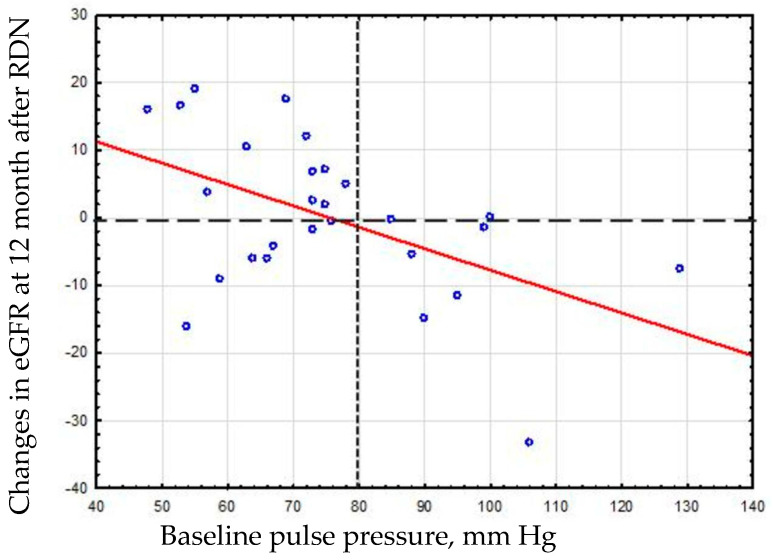
Relationship between baseline PP and eGFR dynamics at 12 months post-dRDN.

Based on the visual analysis of the correlation depicting the relationship between the change in eGFR and PP (Figure 4), the change in eGFR was compared between patient groups with a PP ≥80 mmHg and <80 mmHg. 

The wide dotted line indicates the conditional division of patients with and without nephroprotective effect. The frequent dotted line indicates the conditional division of patients into subgroups stratified by baseline PP (<80 mmHg vs. ≥80 mmHg).

**Figure 5:**
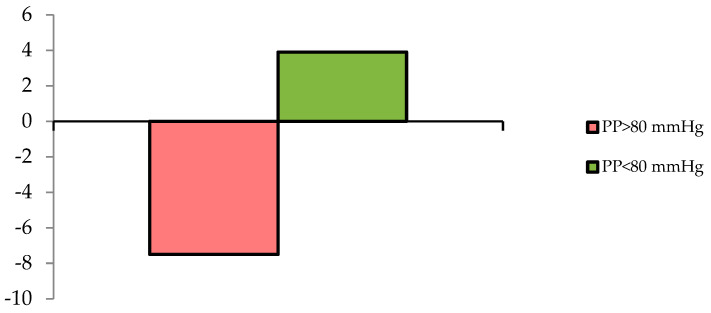
The changes in estimated glomerular filtration rate one year after renal denervation depending on the baseline level of 24 h pulse pressure.

The comparative analysis of eGFR changes between patients with baseline PP ≥ 80 mmHg and <80 mmHg confirmed divergent renal functional trajectories (Figure 5), with a mean intergroup difference of 11.5 mL/min/1.73 m^2^ (95% CI: 2.1–20.6; *p* = 0.018).

Comparative analysis of the nephroprotective effect, defined as the absence of a decline in eGFR, between patients with HbA1c levels ≥8% and <8% revealed significant differences (*p* = 0.037). The frequency of this nephroprotective effect was 66.7% (twelve of eighteen patients) in the HbA1c <8% group, compared to 22.2% (two of nine patients) in the HbA1c ≥8% group. Patients with HbA1c ≥8% have a seven-fold reduction in the likelihood of developing the nephroprotective effect (OR = 0.143, 95% CI: 0.022–0.910). To investigate whether the attenuated nephroprotective effect associated with elevated HbA1c levels was attributable to increased vascular stiffness, PP levels were compared between the HbA1c ≥8% and <8% groups. No significant intergroup differences in PP were detected (77.8 ± 14.8 mmHg vs. 75.4 ± 20.5 mmHg, *p* = 0.741). Similarly, the frequency of elevated PP (>80 mmHg) did not differ significantly between the HbA1c ≥8% and <8% groups (45.5% vs. 27.8%, *p* = 0.282).

## 4. Discussion

This study represents the first evaluation of the ability of the distal RDN to slow or prevent the progressive decline in renal function in diabetic patients with RHTN and renal dysfunction (in most cases). It was a primary finding of this study. No significant changes were observed in serum lipocalin-2, cystatin C, 24 h urinary albumin excretion, renal blood flow parameters, or kidney volumetric measurements during the follow-up period. These findings suggest a clinically meaningful nephroprotective effect. The high between-subject variability of the effect (and other) measurements reflected by the pretty wide 95% CIs may be a specific limitation of the study in terms of the ability to detect the renal effects of distal RDN. Similar nephroprotective efficacy of distal RDN in patients with RHTN was previously demonstrated over a 3 year follow-up [28]; however, in that study, first-generation renal catheters (Symplicity Flex) were used for the intervention. Notably, the mean annual decline in eGFR in T2DM patients is reported as 3.3 ± 8.2 mL/min/1.73 m^2^/year, compared to 2.4 ± 7.7 mL/min/1.73 m^2^/year in non-diabetic individuals [11]. The present analysis found no significant change in eGFR 1 year after dRDN, including in those with CKD. Similar data (on slowing/preventing progression of renal dysfunction after RDN) were reported by Chinese researchers in patients with CKD after a 6 month follow-up [29]. The meta-analysis by Mohammad et al. also demonstrated no progressive decline in renal function in patients with RHTN and CKD after RDN [30]. Data from the global SYMPLICITY registry further reinforced these findings, showing that at 3 years post-RDN, CKD patients exhibited an approximately two-fold slower decline in GFR compared to non-CKD patients (−3.7 mL/min/1.73 m^2^ vs. −7.1 mL/min/1.73 m^2^, respectively) [31], despite comparable reductions in BP.

It is important to emphasize that no significant change in eGFR occurred despite significant BP reduction. At the same time, on the basis of previously conducted studies for hypertensive/diabetic populations, combined disease-related (2–4 mL/min/1.73 m^2^/year) and antihypertensive treatment-associated (2 mL/min/1.73 m^2^) declines—as evidenced by SPRINT and ACCORD-BP trial data—would predict a total annual reduction of 4–6 mL/min/1.73 m^2^.

The analysis of individual data from two large RCTs, SPRINT and ACCORD-BP, has shown that a decrease in BP due to antihypertensive pharmacotherapy causes a proportional decrease in eGFR, starting from a level of ≥10 mm Hg whereas lowering BP to less than 10 mm Hg does not affect eGFR. There was a linear decrease of 3.4% eGFR (95% CI, 2.9–3.9%) per 10 mm Hg mean arterial pressure decrease. The observed eGFR decline based on 95% of the subjects varied from 26% after 0 mm Hg to 46% with a 40 mm Hg mean BP decrease. Accordingly, a decrease in eGFR would be expected in responders, and particularly in super-responders, within our study cohort. Based on the magnitude of 24 h BP reduction achieved, a minimum eGFR decline of 3.4% should have manifested in the overall cohort. The lack of this anticipated GFR reduction points to possible restoration of kidney function, attributable to renal tissue regeneration consequent upon enhanced perfusion and diminished functional burden.

### 4.1. Effects of dRDN on 24 h Ambulatory Blood Pressure

This study documented significant reductions not only in systolic and diastolic BP, but also in PP. The incidence of elevated PP > 60 mm Hg decreased by a factor of 1.9.

The beneficial effect of RDN on PP was also observed in the global SYMPLICITY registry [32], the Regina RDN trial [33] and a meta-analysis of five controlled studies in patients with RHTN at 6 months post-RDN [34].

From an organ protection standpoint, decrease in PP is of considerable clinical importance. Elevated PP reflects increased arterial stiffness, which is associated with adverse cardiovascular [35,36] and renal outcomes [37,38]). As arterial stiffness progresses, the arterial wall’s capacity to dampen pulsatile energy diminishes. Consequently, the renal microvasculature experiences heightened pulsatile stress due to direct transmission of pressure waves to the glomeruli, resulting in barotrauma [39]. This hemodynamic burden initiates a pathophysiological cascade comprising glomerular hypertrophy, hyperfiltration, segmental glomerulosclerosis, and ultimately nephrosclerosis and interstitial fibrosis [37]. Therefore, management strategies for HTN and nephroprotection should encompass comprehensive control of not only systolic and diastolic BP, but also PP.

Additionally, reductions in 24 h and daytime BP variability were observed, consistent with findings from the Persu et al. meta-analysis [40]. It is hypothesized that diminished BP variability may confer independent organ-protective effects by mitigating hemodynamic fluctuations—a significant stressor in the pathophysiology of end-organ damage. In our prior study of patients with diabetes and refractory HTN, RDN reduced nocturnal systolic BP variability [41].

### 4.2. Effects of dRDN on Renal Function and Hemodynamics

No significant changes in cystatin C, NGAL, albuminuria, or MRI-derived renal dimensions were detected, indicating preserved renal structural and functional integrity. These results suggest a good safety profile of the procedure, even among individuals with baseline renal impairment, which is consistent with the data of Liu et al. [29].

No significant changes in albuminuria were observed following renal denervation. Previous studies have shown both a decrease in albuminuria after RDN [42] and the absence of such an effect [33]. Taken together, these data suggest that RDN at least attenuates hypertension-mediated kidney injury and in most cases prevents further albuminuria progression. Data regarding RDN’s effect on cystatin C remain inconsistent [43], which may reflect this biomarker’s dependence on extrarenal factors beyond glomerular function. The finding of unchanged NGAL levels corroborates prior observations by Dörr et al. [44,45]. Renal dimensional and volumetric stability following RDN was previously documented by Sanders et al. [46]. No significant changes in RRI were observed in the overall cohort, CKD/non-CKD subgroups, or when stratified by magnitude of BP reduction. Prior analysis established that RRI decreases in patients with RHTN and T2DM only when baseline elevation exists [47]. Notably, the absence of renal hemodynamic deterioration in hypertensive diabetic patients may reflect nephroprotective effects of the procedure, potentially counteracting persistent adverse metabolic influences. Critically, Al Ghorani et al. reported sustained RRI stability even 10 years post-RDN despite natural vascular aging processes [48]. Conversely, Mahfoud et al. documented significant RRI reductions at 3 and 6 months post-RDN independent of BP lowering magnitude [49]. However, this study enrolled only 15 diabetic patients, limiting generalizability.

### 4.3. Effects of dRDN on Glucose Metabolism and Catecholamines

No alterations in glucose levels or HbA1c were observed. Despite preclinical evidence suggesting renal denervation (RDN) may improve glycemic control through multiple molecular pathways, current clinical trial data remain discordant and do not indicate unequivocal improvement in glucose homeostasis or insulin sensitivity parameters [50]. No changes in plasma or urinary metanephrines were detected, a finding that corroborates results from a recent meta-analysis [51].

### 4.4. Determinants of Renal Function Changes After dRDN

In this study, HbA1c levels and PP were independent determinants of eGFR changes at 12 months post-RDN. Elevated PP can drive irreversible renal damage through chronic barotrauma, where hemodynamic stress is transmitted to the microvasculature and glomerular capillaries. At the same time, post-RDN tonic renal vasodilation and reduced vascular resistance may attenuate arterial wall compliance, potentially facilitating pulsatile energy transmission to glomeruli and exacerbating hemodynamic stress on compromised nephrons. This pathophysiological framework aligns with P. Y. Courand et al.’s findings of progressive eGFR decline in patients with aortic calcification following RDN [52]. Notably, analysis revealed that eGFR reduction occurred predominantly in patients with PP > 80 mmHg. This suggests that RDN’s renoprotective effects may be more pronounced in individuals with minimal vascular stiffness burden. Furthermore, baseline PP > 60 mmHg was previously identified as a risk factor for renal parenchymal volume loss at 12 months post-RDN [53]. Sodium-glucose cotransporter 2 (SGLT2) inhibitors have previously been shown to be effective in slowing CKD progression and reducing cardiovascular risk [54]. It should be noted that patients in this study did not use SGLT2 inhibitors, but all of them received renin–angiotensin–aldosterone system inhibitors.

### 4.5. Study Limitations

This work is a preliminary pilot study designed primarily to assess the safety and feasibility of the procedure. The present data are not intended to support clinical decisions. Instead, this study provides foundational safety information that will guide the design of future controlled trials comparing treated versus sham groups to more rigorously evaluate clinical efficacy.

The notable strengths of this study include the use of a second-generation multi-electrode catheter (Symplicity Spyral™), assessment of BP reduction via ambulatory blood pressure monitoring (ABPM), the center’s extensive experience in performing renal denervation (RDN) procedures, and the absence of confounding due to SGLT2 inhibitor therapy during the study.

Several important study limitations should be acknowledged. First, this was a single-arm study with no sham-control group. The lack of blinding and a control arm could introduce expectancy bias. However, several rigorously controlled studies of RDN have previously shown a quite insignificant effect of the sham procedure on BP at 3–6 months after the intervention. Second, the small sample size may not have provided enough power to detect some between-group differences in secondary outcomes. Third, medication adherence was assessed through patient self-report. Fourth, potential confounding from variable glycemic control and evolving medication regimens during follow-up cannot be ruled out.

### 4.6. Perspectives

The potential benefits of RDN’s sympatholytic effect warrant further investigation and may provide therapeutic advantages for other conditions characterized by heightened sympathetic activity, such as heart failure. Given that SGLT2 inhibitors reduce sympathetic nervous system activity [55,56,57,58,59], improve metabolic control, and confer nephroprotective effects, future randomized studies are needed to examine the impact on renal function of combining RDN with SGLT2 inhibitors in patients with RHTN and type T2DM.

Another promising research avenue involves exploring the nephroprotective potential of combining RDN with the non-steroidal mineralocorticoid receptor antagonist finerenone, which has demonstrated robust evidence for reducing risks of CKD progression and cardiovascular complications [60]. Undoubtedly, findings in this study require further validation in larger randomized controlled trials. As evidence accumulates supporting the nephroprotective efficacy of RDN, this procedure may emerge as the fourth pillar of therapeutic strategies to retard kidney disease progression in diabetes—alongside renin–angiotensin–aldosterone system blockade, SGLT2 inhibitors, and finerenone [61].

Given that CKD patients are at high risk of contrast-induced nephropathy following contrast-enhanced RDN procedures [32], which may also limit the realization of the intervention’s nephroprotective potential, future research could focus on comparing changes in renal function after RDN is performed with contrast-based versus non-contrast CO_2_ angiography [62].

## 5. Conclusions

In conclusion, distal renal denervation in patients with RHTN and concomitant T2DM is safe and associated not only with significant and sustained BP reduction but also with the stabilization of renal function in contrast to the expected decrease in that function from the natural decline in patients with HTN or diabetes and, also, as a result of the BP lowering effect of RDN. This nephroprotective effect of distal RDN in patients with RHTN and T2DM is negatively affected by arterial stiffness and hyperglycemia at baseline, which may be partly attributable to treatment-induced reductions in arterial stiffness.

## Figures and Tables

**Table 1:** Patient baseline characteristics, [M ± SD, n (%)].

Measurement	n = 29
Age, years	61.6 ± 7.2
Sex, male	10 (34.5)
Body mass index, kg/m^2^	35.7 ± 5.5
Abdominal obesity	27 (93.1)
General obesity	27 (93.1)
Known duration of hypertension, years	21.4 ± 10.6
Known duration of diabetes mellitus, years	13.3 ± 8.4
Coronary artery disease	23 (79.3)
History of myocardial infarction	7 (24.1)
History of stroke	4 (13.8)
Peripheral artery disease	28 (96.6)
Dyslipidemia	27(93.1)
Left ventricular hypertrophy	20 (69.0)
Left ventricular ejection fraction, %	66.6 ± 4.0
Office systolic BP/diastolic BP, mmHg	165.6 ± 24.3/89 ± 18.3
Office heart rate, bpm	68.1 ± 9.3
24 h systolic BP/diastolic BP, mmHg	158.1 ± 21.4/81.8 ± 12.4
24 h pulse pressure, mmHg	76.3 ± 18.3
24 h heart rate, bpm	64.7 ± 7.3
HbA1c, %	7.8 ± 1.3
Fasting plasma glucose, mmol/L	8.4 ± 3.2
eGFR, mL/min/1.73 m^2^	56.7 ± 19.9
Chronic kidney disease stage	21 (72.4)
● G3 A1 ● G3 A2 ● G3 A3	14 (48.2) 6 (20.6) 1 (3.4)
Isolated albuminuria	
● G1 A3 ● G2 A3	1 (3.4) 1 (3.4)
Isolated systolic hypertension	16 (55.2)
Patients with high pulse pressure (>60 mm Hg)	23 (79.3)

**Table 2:** Characteristics of antihypertensive and antidiabetic therapy, n (%).

Number of antihypertensive drugs	4.4 ± 1.0
Beta blockers	27 (93.1)
Angiotensin-converting enzyme inhibitors/angiotensin receptor blockers	27 (93.1)
Diuretics	29 (100)
Calcium antagonists	25 (86.2)
Spironolactone	10 (34.5)
I1-Imidazoline receptor agonists (moxonidine)	5 (17.2)
Alpha blocker (doxazosin)	9 (31.0)
Glucose-lowering medications	
Oral hypoglycemic monotherapy	9 (31.0)
Combined oral hypoglycemic therapy	8 (27.6)
Insulin + Oral hypoglycemic agents	12 (41.4)

**Table 3:** Change in secondary outcome and renal function compared to baseline in responders and non-responders (n = 27). Data are mean and 95% confidence interval.

**Laboratory Data**	
24 h urinary albumin excretion, mg/24 h	194.7 [−263.9; 653.2]
Lipocalin (NGAL), ng/mL	8.9 [−12.3; 29.9]
Cystatin C, mg/L	−123.3 [−379.2; 132.7]
**MRI Kidney**	
The volume of the kidney (right/left mL^3^)	−11.1 [−42.1; 19.9]/4.5 [−34.8; 25.9]
Renal cortex volume of the right kidney, mL^3^	−4.8 [−13.9; 4.4]
Renal cortex volume for the left kidney, mL^3^	−0.8 [−9.1; 7.7]
The medulla volume for the right kidney, mL^3^	−1.0 [−24.7; 22.9]
The medulla volume for the left kidney, mL^3^	0.8 [−25.9; 27.3]
**Renal Artery Doppler Ultrasound**	
RRI in the main trunk of RA	−0.06 [−0.14; 0.03]
RRI for segmental RA	−0.05 [−0.13; 0.04]
Peak blood flow velocity in the trunk of RA	−4.4 [−11.4; 2.7]
Peak blood flow velocity in segmental RA	−6.2 [−20.6; 8.3]

**Table 4:** Change in BP and renal function compared to baseline in responders and non-responders. Data are mean and 95% confidence interval.

	Responders (n = 17)		Non-Responders (n = 10)	
	Δ Follow-Up	*p*	Δ Follow-Up	*p*
**Laboratory Data**				
eGFR, mL/min/1.73 m^2^	−0.6 [−14.9; 13.7]	0.934	5.1 [−12.6; 22.7]	0.551
24 h urinary albumin excretion, mg/24 h	−52.1 [−134.5; 30.4]	0.207	−83.2 [−250.2; 84.0]	0.310
Lipocalin-2 (NGAL), ng/mL	14.2 [−15.8; 44.1]	0.341	4.3 [−19.1; 27.7]	0.703
Cystatin C, mg/L	−132.8 [−519.2; 253.6]	0.479	−111.9 [−499.6; 275.8]	0.539
**MRI Kidney**				
The volume of the kidney (right/left mL^3^)	−14.1 [−52.8; 24.7]/ −10.4 [−54.2; 33.5]	0.460/0.630	−4.4 [−64; 55.3]/ 4.1 [−40.5; 48.6]	0.878/0.847
Renal cortex volume of the right kidney, mL^3^	−2.7 [−14.1; 8.8]	0.634	−7.8 [−25.2; 9.6]	0.351
Renal cortex volume for the left kidney, mL^3^	−3.3 [−14.3; 7.8]	0.547	2.8 [−11.6; 17.0]	0.687
The medulla volume for the right kidney, mL^3^	−3.8 [−34.2; 26.6]	0.798	4.2 [−40.6; 48.9]	0.846
The medulla volume for the left kidney, mL^3^	−7.1 [−45.6; 31.6]	0.710	12.3 [−25.3; 49.8]	0.494
**Renal Artery Doppler Ultrasound**				
RRI in the main trunk of RA	−0.1 [−0.3; 0.1]	0.141	−0.02 [−0.10; 0.06]	0.581
RRI for segmental RA	−0.2 [−0.3; 0.1]	0.104	−0.02 [−0.09; 0.06]	0.695
Peak blood flow velocity in the trunk of RA	−11.2 [32.6; 10.2]	0.295	2.4 [−11.1; 15.9]	0.716
Peak blood flow velocity in segmental RA	−4.5 [−13.9; 4.9]	0.337	−4.3 [−15.7; 7.2]	0.442

**Table 5:** Change in glucose metabolism status and laboratory markers of sympathetic nervous system activity compared to baseline in responders and non-responders. Data are mean and 95% confidence interval.

	All Patients (n = 27)	Responders (n = 17)		Non-Responders (n = 10)	
	Δ Follow-Up	Δ Follow-Up	*p*	Δ Follow-Up	*p*
FPG, mmol/L	−0.5 [−2.0; 1.0]	−0.5 [−2.4; 1.5]	0.662	−0.7 [−3.4; 2.1]	0.614
HbA1c, %	0.1 [−0.7; 0.9]	−0.1 [−0.9; 0.8]	0.935	0.4 [−1.6; 2.2]	0.723
Plasma metanephrine, ng/mL	2.9 [−15.4.21.0]	10.6 [−11.8; 32.9]	0.341	−11.7 [−46.2; 22.9]	0.483
Plasma normetanephrine, ng/mL	−41.5 [−132.8; 50.0]	−93.8 [−220.8; 33.3]	0.142	57.8 [−32.2; 147.7]	0.191
24 h urinary metanephrine excretion, mg/24 h	14.6 [−48.8; 77.9]	6.9 [−80.4; 94.1]	0.874	32.4 [−56.7; 121.4]	0.453
24 h urinary normetanephrine excretion, mg/24 h	−27.0 [−162.5; 108.7]	−37.3 [−245.4; 170.9]	0.718	−4.4 [−117.5; 108.9]	0.937

**Table 6:** Multivariate regression analysis of factors influencing eGFR dynamics at 1 year post-dRDN.

Variable	β Coefficients	Standard Error	*p*
Baseline 24 h ambulatory pulse pressure	−0.2414	0.1015	0.0269
HbA1c	−4.8967	1.7336	0.0102
Age	−0.1390	0.2767	0.6208
Baseline 24 h ambulatory systolic BP	−0.0423	0.1143	0.7153
Male	−5.5411	4.0854	0.1894

## Data Availability

Data are available upon reasonable request from the corresponding author.

## Abbreviations

The following abbreviations are used in this manuscript:

24 h SBP	24 h ambulatory systolic blood pressure
BP	Blood pressure
BPM	Beats per minute
CKD	Chronic kidney disease
DBP	Diastolic blood pressure
dRDN	Distal renal denervation
eGFR	Estimated glomerular filtration rate
HbA1c	Glycosylated hemoglobin
HTN	Hypertension
MRI	Magnetic resonance imaging
RHTN	Resistant hypertension
RRI	Renal resistive index
SBP	Systolic/diastolic blood pressure
SNS	Sympathetic nervous system
T2DM	Type 2 diabetes mellitus
